# 

**Published:** 1898-08

**Authors:** 


### Southern Medical Record

A Monthly Journal of Medicine and Surgery.

VOL. XXVIII. ATLANTA, GA., AUGUST, 1898. No. 8

BERNARD WOLFF, M. D., EDITOR.

ASSOCIATE EDITORS:

A. W. GRIGGS, M. D.
J. McFADDEN GASTON, M. D.
J. McFADDEN GASTON, Jr., M. D.
WILLIS F. WESTMORELAND, M. D
B. M. ZETTLER, BUSINESS MANAGER.

